# Development of a dendritic cell-targeted vaccine strategy using proximity-induced conjugation

**DOI:** 10.7150/thno.122332

**Published:** 2026-02-11

**Authors:** Zhidong Wang, Xiaolin Yang, Jianjiang Li, Guang Chen, Haodi Ma, Zhengshuang Xu, Yu J. Cao

**Affiliations:** 1 State Key Laboratory of Chemical Oncogenomics, Shenzhen Key Laboratory of Chemical Genomics, Peking University Shenzhen Graduate School, Shenzhen, Guangdong, 518055, China.; 2 Institute of Chemical Biology, Shenzhen Bay Laboratory, Shenzhen, 518132, China.

**Keywords:** dendritic cell-targeted vaccine, proximity-induced conjugation, unnatural amino acids, neoepitopes, immune checkpoint inhibitor

## Abstract

**Background:**

Traditional cancer vaccines that utilize peptides or proteins often exhibit limited efficacy as a result of mutations in cancer antigenic epitopes, also known as antigenic drift, which reduce the ability of traditional vaccines to target tumor antigens and elicit robust immune response.

**Methods:**

To address these challenges, we propose an innovative and universal strategy for dendritic cell (DC)-targeted neoepitope delivery via proximity-induced conjugation (PIC). This approach enables the site-specific crosslink of a broad spectrum of neoepitopes tailored to diverse cancer types, thereby increasing both vaccine flexibility and applicability. The PIC method involves the use of recombinant Fc-affinity peptides that are modified with two distinct unnatural amino acids: the photoreactive amino acid p-benzoyl-L-phenylalanine (pBPA) and the bioorthogonal reactive amino acid 4-fluorophenyl carbamate lysine (FPheK). These modified peptides allow for the precise conjugation of neoepitopes through ultraviolet (UV) irradiation or mild incubation, thereby achieving controlled antigen coupling.

**Results:**

Through optimization of this strategy, we observed a substantial increase in DCs mediated antigen uptake and processing, leading to enhanced T cell activation, a robust cytotoxic immune response, and significant improvements in antitumor efficacy. Moreover, the DC-targeted vaccine exhibited promising synergistic effects with an immune checkpoint inhibitor (ICI), resulting in a marked reduction in tumor growth and prolonged survival in preclinical models.

**Conclusion:**

These findings underscore the potential of the PIC-based DC-targeted vaccine system to augment the immunogenicity, versatility, and therapeutic efficacy of cancer vaccines. This strategy offers a compelling solution to the challenges posed by antigenic drift and mutation, thereby improving clinical outcomes across a broad range of cancers.

## Introduction

Immunotherapy has revolutionized the approach of cancer treatment over the past two decades, resulting in transformative breakthroughs in the fight against this disease 1. Notably, adoptive T-cell therapy has proven to be highly effective among these innovations, particularly in treating hematologic malignancies 2, 3. However, progress in treating solid tumors successfully with these therapies has been more gradual and challenging. Cancer vaccines, another promising class of immunotherapies, are designed to prime the patient's immune system to precisely recognize and eliminate cancer cells, and significant efforts are therefore to develop such vaccines for improving the treatment of solid tumors 4. To date, the FDA has approved two cancer vaccines: Provenge for refractory prostate cancer and Gardasil-9 for cervical cancer 5, 6. Despite these achievements, the clinical efficacy of tumor vaccines in solid tumors remains limited because of their inherently low immunogenicity and challenges in effectively presenting antigens to antigen-presenting cells (APCs) 7. To overcome these barriers, targeting dendritic cells (DCs) for antigen delivery has emerged as a highly effective way to increase the immunogenicity of cancer vaccines, offering new hope for improving solid tumor treatment outcomes 8.

Conventional dendritic cells (cDCs) play an essential role in immune activation by stimulating both CD8⁺ and CD4⁺ T cells through the MHC class I and II pathways 9. Notably, increased infiltration of cDC1s into tumors is correlated with improved patient outcomes 10. DCs primarily capture antigens through C-type lectin receptors (CLRs), which recognize pathogen-associated molecular patterns, triggering endocytosis and antigen processing for peptide-MHC complex presentation 11. In recent studies, monoclonal antibodies have been used to promote antigen uptake and processing by exploiting this endocytic pathway 12, 13. CD205 (DEC-205) 14, a key CLR, has shown remarkable antigen-presenting ability, prompting researchers to utilize CD205-targeted antibodies for delivering tumor-associated and viral antigens (e.g., OVA, HIV gag p24, NY-ESO-1, and EBNA1) via direct genetic fusion 15-18. However, conventional fusion methods may compromise anti-CD205 antibody functionality and limit the full modular potential of antibody-mediated antigen delivery platforms. More critically, there is an urgent need for innovative vaccine design and delivery systems to create more effective, off-the-shelf cancer vaccines.

Antibodies serve as versatile carriers for the targeted delivery of bioactive molecules, including small-molecule drugs, nucleic acids, radioisotopes, and proteins 19, 20. To increase the flexibility of antibody-based delivery platforms, three strategies have been developed: modular assembly, chemical modifications, and unnatural amino acid (UAA)-mediated conjugation 21-25. While these approaches all enable covalent or noncovalent attachment, diverse conjugation options, and precise site-specific labeling, UAA-based methods offer superior control over drug-to-antibody ratios (DARs) and conjugation specificity with minimal immunogenicity and functional disruption. Notably, the proximity-induced conjugation (PIC) strategy stands out for its simplicity, requiring only a single UAA (e.g., sulfotyrosine) in the antibody or cargo to enable covalent linkage within proximal residues 26, 27. As a result, the PIC platform has demonstrated remarkable versatility and has been effectively utilized in the production of multivalent antibodies, covalent protein drugs, antibody‒drug conjugates (ADCs), and antibody‒based probes 28-30. This broad applicability highlights its significant potential in advancing precision therapeutics.

Here, we generated a DC-targeted vaccine delivery system with enhanced adaptability and efficiency using photoreactive PIC (P-PIC) and chemically reactive PIC (C-PIC). This vaccine system is composed of an anti-CD205 antibody as the targeting module, coupled with Fc-affinity peptides modified with either p-benzoyl-L-phenylalanine (pBPA) or 4-fluorophenyl carbamate lysine (FPheK) to facilitate precise PIC-mediated conjugation of neoepitopes derived from OVA or LMP2A. This approach significantly increases antigen uptake by DCs *in vitro* and promotes T-cell activation and potent antitumor responses *in vivo*. While the OVA-derived epitope vaccine demonstrated synergistic antitumor effects when combined with a PD-1 inhibitor, the vaccine carrying the LMP2A-derived epitope enrichment region (EER) could elicit strong T-cell responses and improve survival outcomes, even in the absence of PD-1 blockade, highlighting the adaptability and broad applicability of DC-targeted vaccines in antigen delivery. Overall, our study establishes a robust platform for universal DC-targeted vaccines, demonstrating the potential of PIC-based strategies to increase vaccine immunogenicity while significantly reducing production time.

## Results

### Optimization of P-PIC for DC-targeted vaccine antigen delivery

**Figure 1:**
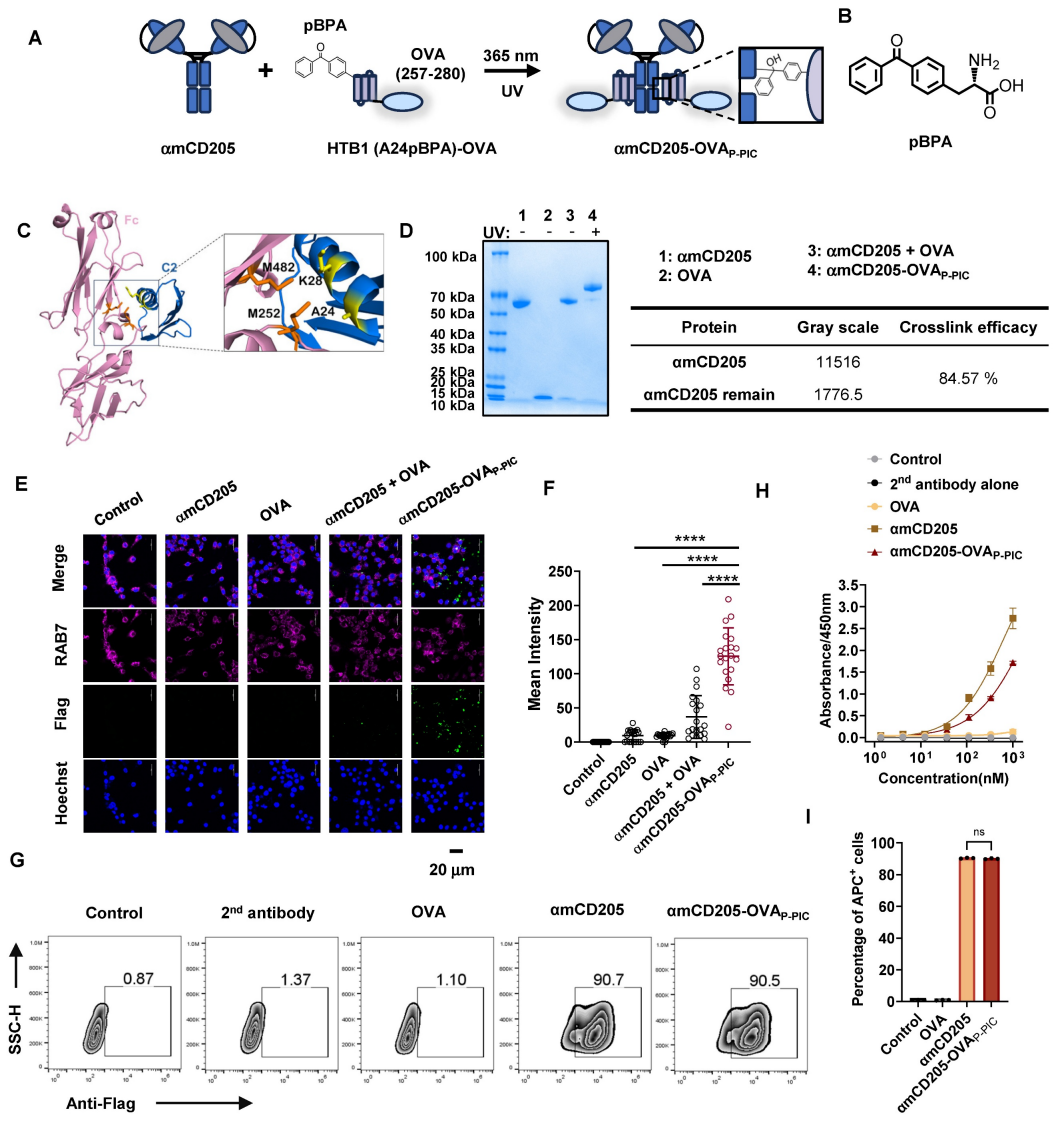
** P-PIC increases antigen uptake *in vitro*.** (A) Schematic representation of P-PIC. The ovalbumin epitope OVA (257-280) was fused to HTB1, with pBPA incorporated at the A24 site. Covalent conjugation was induced upon exposure to 365 nm UV light. (B) Structure of pBPA. (C) Structure of IgG1 Fc fragment with its adaptor. HTB1 is a variant of protein G C2 domain. PDB file: 1FCC. (D) Reducing SDS-PAGE analysis of αmCD205, OVA, and mixed proteins with or without UV exposure. (E) Colocalization images of RAW264.7 cells incubated with αmCD205, OVA, αmCD205 + OVA, or αmCD205-OVA_P-PIC_. The vaccines were stained with an anti-Flag antibody (green), RAB7 was stained with an anti-RAB7 antibody (pink), and the nuclei were stained with Hoechst (blue). Scale bars = 20 μm. (F) Quantification of the mean integrated fluorescence intensity in the indicated groups (n = 20). (G) Representative flow cytometry plots showing the binding of OVA, αmCD205 and αmCD205-OVA_P-PIC_ to RAW264.7 cells. (H) Cell-based ELISA result of binding affinity of OVA, αmCD205 and αmCD205-OVA_P-PIC_ to RAW264.7 cells (n = 3). (I) Quantification of flow cytometry binding (n = 3). The data are shown as the means ± SDs. Statistical significance was determined using a one-way ANOVA with Tukey's multiple comparisons test (* p < 0.05, ** p < 0.01, *** p < 0.001, **** p < 0.0001).

A critical challenge in developing DC-targeted strategies is the identification and optimization of highly effective targeting molecules. To address this, we selected CD205 as the target and engineered its single chain fragment variable (scFv) (Clone: NLDC-145), and systematically evaluated the optimal orientation of the light and heavy chains. We compared the binding affinities of two constructs, αmCD205-scFv-HL and αmCD205-scFv-LH (Figure S1A-B), in a mouse CD205^+^ macrophage line. The αmCD205-scFv-LH construct exhibited superior binding affinity (Figure S1C-D), which was further confirmed by cell-based ELISA (Figure S1E). To enhance its functionality, we fused the optimized αmCD205-scFv-LH to a human IgG1 Fc fragment, serving as a conjugation handle, thereby generating the full-length construct αmCD205. Unless otherwise specified, αmCD205 refers to the anti-mouse CD205-scFv-LH-Fc fusion protein (Figure S2). To facilitate targeted antigen epitope delivery, we optimized a P-PIC strategy, enabling site-specific and efficient epitope conjugation onto the antibody (Figure 1A). For this purpose, we employed HTB1, a natural Fc-affinity peptide 29, which incorporates p-benzoyl-L-phenylalanine (pBPA) (Figure 1B), and fuses it with the OVA epitope (257-280) 31. Previous studies have indicated that residues A24 and K28 within HTB1 are positioned near M252 and M482 of IgG1 Fc (Figure 1C), making them ideal sites for UAA incorporation 30. Accordingly, we constructed and expressed HTB1 (A24pBPA)-OVA and HTB1 (K28pBPA)-OVA and verified their molecular weights via LC-MS analysis (Figure S3). Upon exposure to 365 nm UV light, a site-specific covalent bond formed between αmCD205 and HTB1, with HTB1 (A24pBPA)-OVA demonstrating superior conjugation efficiency compared with its K28pBPA counterpart (Figure S4).

In light of these results, we selected A24 of HTB1 for pBPA incorporation in the development of a P-PIC-based DC-targeted antigen delivery system. Comparison of affinity to αmCD205 between wild-type and HTB1 (A24pBPA)-OVA proteins revealed that incorporating pBPA at position A24 minimally affected the binding affinity (Figure S5). Optimization of the conjugation protocol revealed that an 8:1 molar ratio of HTB1 (A24pBPA)-OVA to αmCD205, followed by 365 nm UV exposure for 2 h, achieved over 90% conjugation efficiency (Figure S6A-D and Figure 1D). Importantly, antigen integrity was preserved, with no detectable molecular weight loss after 2h of UV exposure (Figure S7). The efficiency of antigen uptake was assessed in RAW264.7 macrophages via immunofluorescence staining. The colocalization of the endosome marker RAB7 with the antigen epitope indicated significantly greater uptake in the conjugated group (αmCD205-OVA_P-PIC_) than in the mixed group (αmCD205 + OVA) (Figure 1E-F) 32. Flow cytometry further confirmed the superior antigen-binding efficiency of the conjugated vaccine in RAW264.7 macrophages (Figure 1G). To extend the applicability of this approach, we developed a human DC-targeted antigen delivery system using an anti-human CD205 antibody (Clone: MG38-3) 33. MG38-3-scFv-LH was fused to an IgG1 Fc fragment to generate αhCD205, which was expressed and subjected to P-PIC following the same procedure (Figure S8A-B). Immunofluorescence staining of THP-1 confirmed significantly increased antigen uptake upon conjugation, closely mirroring the results obtained with a mouse DC-targeted system (Figure S6C-D). The binding efficiency of αhCD205-OVA_P-PIC_ in THP-1 cells coincided with its colocalization results, with 99% binding efficiency (Figure S8E-G). Overall, our optimized DC-targeted antigen delivery system, which is applicable to both mouse and human models, significantly increases antigen uptake *in vitro*, providing a promising platform for improving antigen presentation and eliciting robust immune responses in next-generation DC-targeted vaccines.

### DC-targeted OVA vaccine exhibits enhanced immunogenicity and tumor-suppressing ability

**Figure 2:**
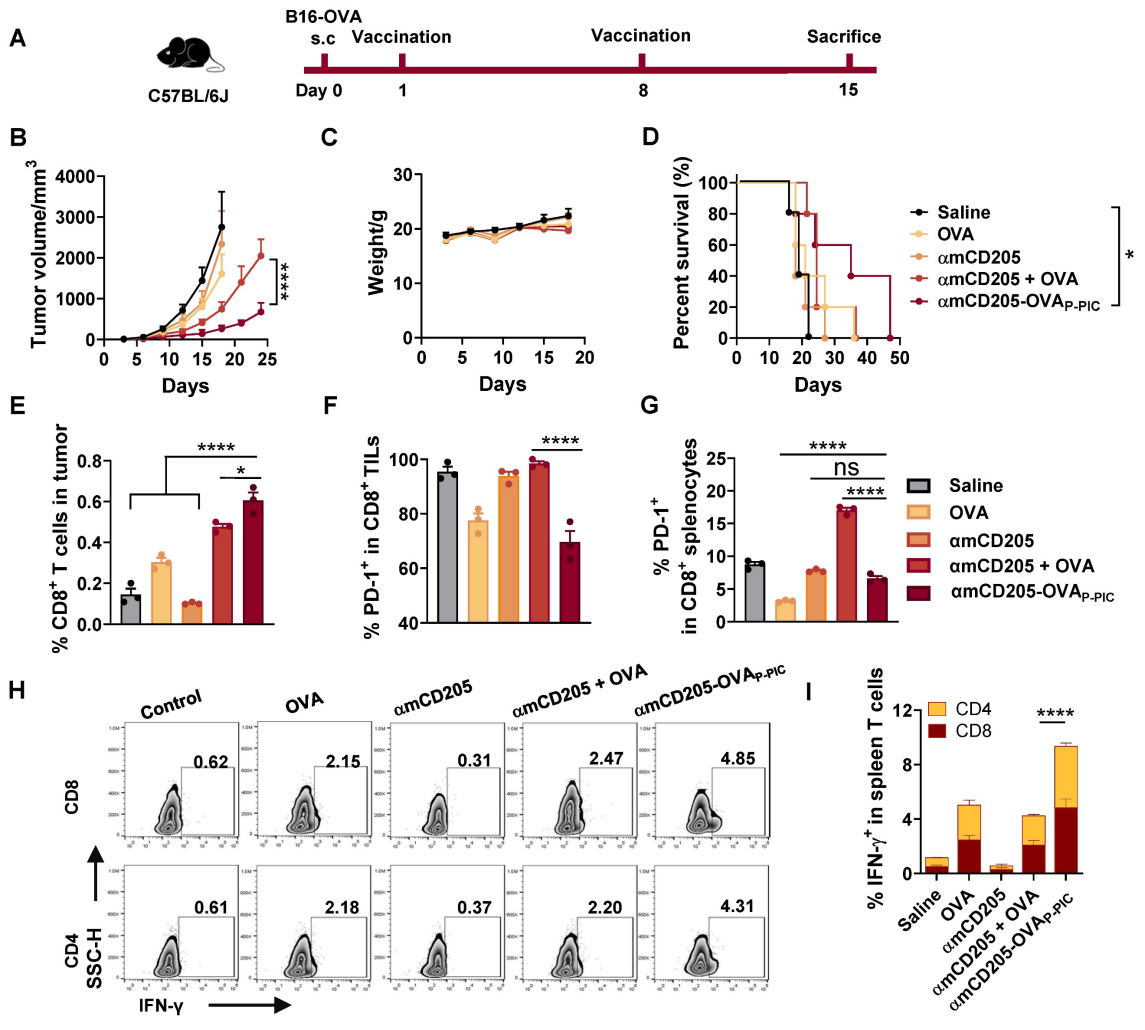
** P-PIC-mediated OVA epitope delivery resulted in tumor growth inhibition *in vivo*.** (A) Schematic representation of the B16-OVA therapeutic model timeline. (B) Tumor growth curves. (C) Mouse body weight data. (D) Survival curves of the mice in the indicated groups (n = 8). (E) Analysis of tumor-infiltrating CD8^+^ lymphocytes and (F) PD-1 expression in CD8^+^ TILs. (G) PD-1 expression levels in splenic CD8^+^ T cells (n = 3). (H) Representative intracellular cytokine staining images of splenocytes showing IFN-γ-positive staining in CD8^+^ and CD4^+^ T cells. (I) Quantification of IFN-γ expression in CD8^+^ and CD4^+^ splenic T cells (n = 3). The data are shown as the means ± SDs. Statistical significance was determined using a one-way ANOVA with Tukey's multiple comparisons test for (E-G), two-way ANOVA with Tukey's multiple comparisons test for (B) and (I), and log-rank test for (D) (* p < 0.05, ** p < 0.01, *** p < 0.001, **** p < 0.0001).

To assess the efficacy of DC-targeted antigen delivery, we established a B16-OVA melanoma model in C57BL/6 mice. The mice were subcutaneously injected with 0.5 × 10^6^ B16-OVA cells and received intraperitoneal priming and booster vaccination on days 1 and 8 (Figure 2A). The experimental groups included the saline, OVA (HTB1 (A24pBPA)-OVA), αmCD205, αmCD205 + OVA (noncovalent mixture) and αmCD205-OVA_P-PIC_ (covalently conjugated vaccine via P-PIC) groups. As shown in Figure 2B, αmCD205-OVA_P-PIC_ significantly suppressed tumor growth and extending survival with no effects on body weight (Figure 2C-D). Seven days after booster vaccination, tumor-infiltrating lymphocytes (TILs) were analyzed, revealing a marked increase in CD8^+^ T-cell infiltration in the αmCD205-OVA_P-PIC_-treated group (Figure 2E), whereas CD4^+^ TIL levels remained unchanged (Figure S9A). Notably, PD-1 expression was selectively reduced on tumor-infiltrating and splenic CD8^+^ T cells (Figures 2F-G, S9B), suggesting decreased T-cell exhaustion. Mechanistically, covalent conjugation likely increased DC-targeted uptake and facilitated rapid antigen clearance, whereas the noncovalent αmCD205 + OVA mixture resulted in prolonged antigen exposure due to continuous binding and dissociation. It has been established that extended antigen persistence in the circulation can induce immune tolerance 34, 35, which may explain the elevated PD-1 expression observed in the mixed group compared with the conjugated group. Furthermore, αmCD205-OVA_P-PIC_ treatment significantly increased the number of IFN-γ^+^ T cells in the splenocytes, which was correlated with enhanced tumor suppression (Figure 2H-I). ELISA of serum OVA-specific antibody titers suggested a minimal contribution of humoral immunity to tumor inhibition in this model (Figure S9C). This is likely attributable to intracellularly expression of OVA in B16-OVA tumor cells, which precludes surface antigen recognition and thereby limits both antibody binding and antibody-dependent cellular cytotoxicity. Together, this finding highlights the potential of PIC-based DC-targeted antigen delivery to enhance tumor-specific immunity and effectively suppress tumor growth.

### Synergistic therapeutic efficacy of DC-targeted vaccines and immune checkpoint inhibitors (ICIs)

**Figure 3:**
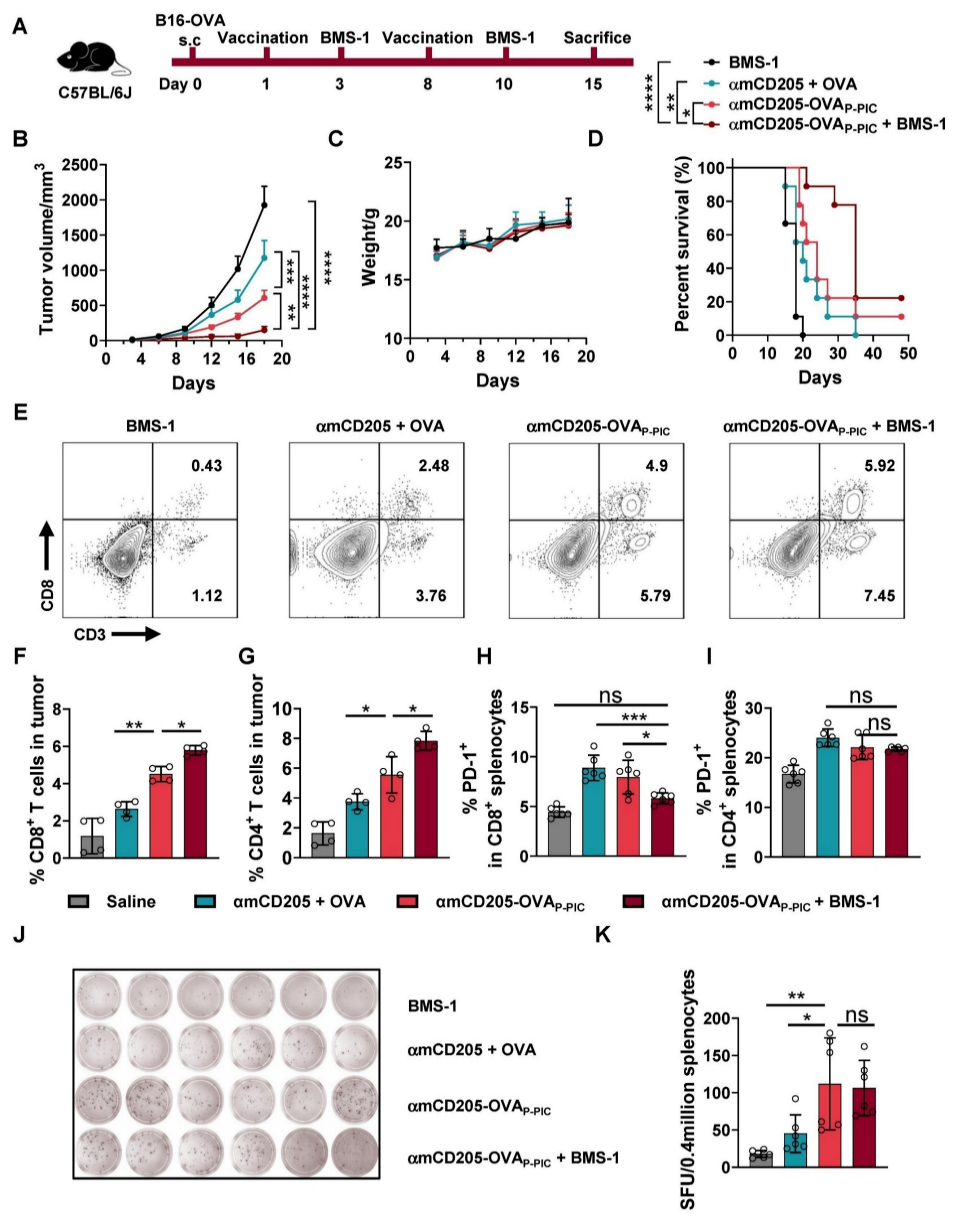
** Synergistic therapy with DC-targeted vaccines and ICIs prolonged survival and alleviated T-cell exhaustion.** (A) Schematic representation of the B16-OVA therapeutic model timeline. BMS-1 was administered two days after each vaccination. (B) Tumor growth curves (n = 6). (C) Mice body weight data. (D) Survival curves of the mice in the indicated groups (n = 9). (E) Representative plots of TIL detection. TILs were divided into CD3^+^ CD8^+^ and CD3^+^ CD8^-^ populations. (F) Quantification of CD8^+^ T cells and (G) CD4^+^ T cells in tumor tissue (n = 4). (H) PD-1 expression levels in splenic CD8^+^ T cells and (I) CD4^+^ T cells on day 15 (n = 6). (J) Representative images of IFN-γ ELISpot assays in the indicated groups (n = 6). (K) Summary of ELISpot counts. SFU: spot-forming units. The data are shown as the means ± SDs. Statistical significance was determined using a one-way ANOVA with Tukey's multiple comparisons test for (F-I) and (K), two-way ANOVA with Tukey's multiple comparisons test for (B) and log-rank test for (D) (* p < 0.05, ** p < 0.01, *** p < 0.001, **** p < 0.0001).

To enhance antitumor efficacy, we combined the DC-targeted vaccine with PD-1/PD-L1 blockade using BMS-1 (an anti-PD-1 inhibitor) 36, which was administered intraperitoneally two days after each vaccination (Figure 3A). This synergistic therapy significantly inhibited B16-OVA tumor growth and prolonged the survival of the mice in the combination treatment group compared with the BMS-1, αmCD205 + OVA and αmCD205-OVA_P-PIC_ groups, with only modest changes in body weight (Figure 3B-D). TIL analysis revealed that combination therapy resulted in a substantial increase in both CD8^+^ and CD4^+^ T-cell infiltration within treated tumors (Figure 3E-G). Additionally, PD-1 expression in splenic CD8^+^ T cells was significantly lower in the combination therapy group than in the αmCD205 + OVA and αmCD205-OVA_P-PIC_ groups (Figure 3H-I), suggesting a reduction in T-cell exhaustion and increased immune activity. ELISpot assays were conducted to assess cellular immunity and revealed that the αmCD205-OVA_P-PIC_ and combination therapy groups generated more spots than the αmCD205 + OVA and BMS-1 groups did (Figure 3J‒K). Overall, the combination of BMS-1 and αmCD205-OVA_P-PIC_ not only increased CD8^+^ and CD4^+^ T-cell infiltration into the tumor but also alleviated immune tolerance, resulting in significant tumor suppression. This highlights the potential of combining ICIs with DC-targeted vaccines to stimulate more robust antitumor immune responses and improve therapeutic outcomes.

### Exploration of DC-targeted delivery of the EER

**Figure 4:**
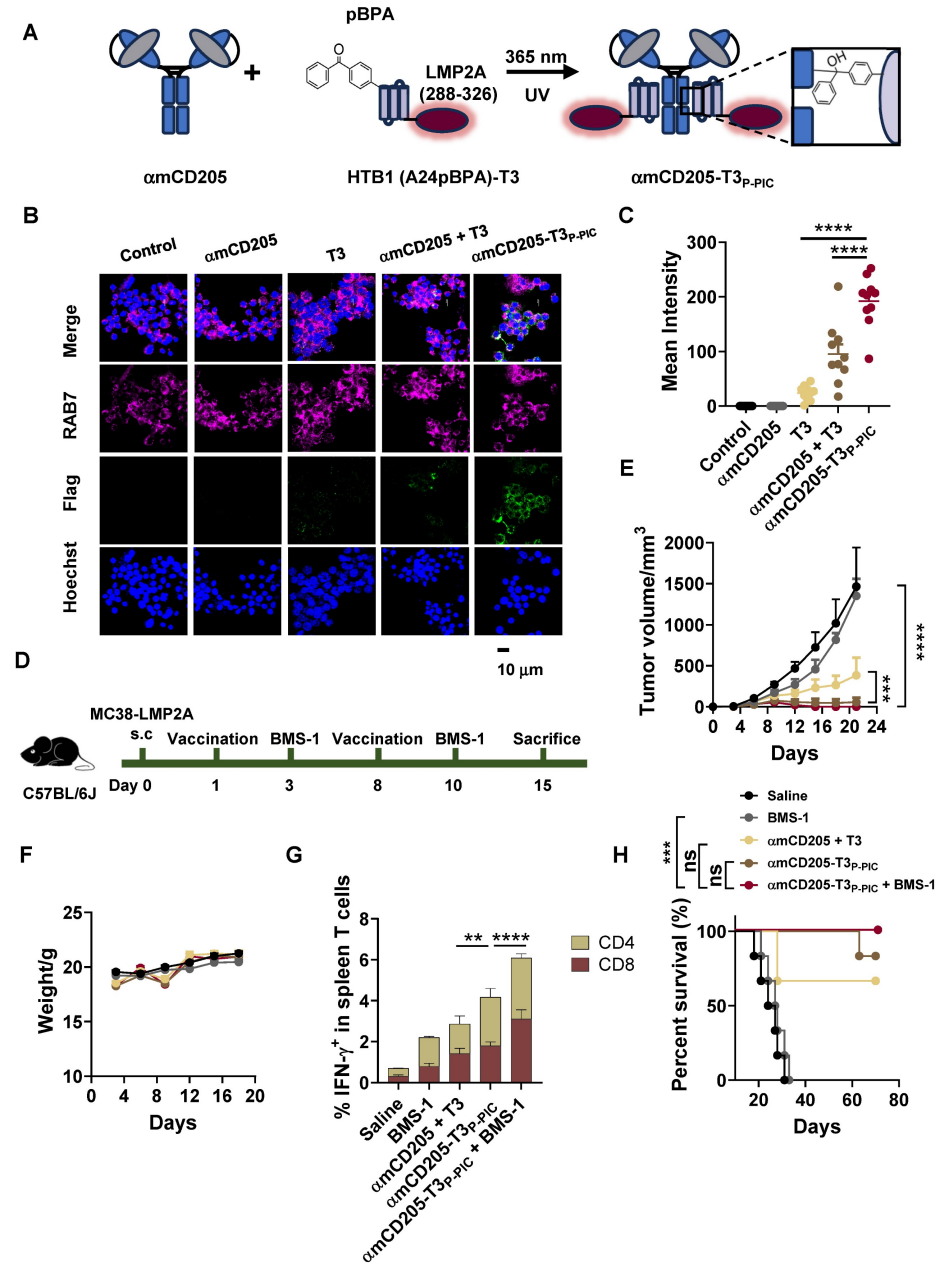
** Exploration of DC-targeted delivery of the T3 EER.** (A) Schematic representation of T3 conjugation to αmCD205 via P-PIC. LMP2A (288-326) was fused to HTB1, with pBPA incorporated at the A24 site. Covalent conjugation was induced upon exposure to 365 nm UV light. (B) Representative colocalization images of RAW264.7 cells incubated with the indicated proteins. Scale bars = 10 μm. (C) Quantification of the mean integrated fluorescence intensity of the indicated proteins (n = 10). (D) Schematic timeline of P-PIC vaccine administration in the MC38-LMP2A therapeutic model. (E) Tumor growth curves and (F) mouse body weights were monitored every three days (n = 9). (G) Quantification of IFN-γ^+^ T cells in the splenocyte population (n = 3). (H) Survival curves of the mice treated with the corresponding vaccines or saline (n = 6). The data are shown as the means ± SDs. Statistical significance was determined using a one-way ANOVA with Tukey's multiple comparisons test for (C). For (E) and (G), two-way ANOVA with Tukey's multiple comparisons test was used. Survival curve was analyzed with log-rank test (* p < 0.05, ** p < 0.01, *** p < 0.001, **** p < 0.0001).

To expand the potential applications of the DC-targeted delivery system, we leveraged this strategy to deliver an EER derived from our previous research, designated EER T3 37. The localization of T3 within LMP2A and its functional epitopes is illustrated in Figure S10. For DC-targeted delivery, the HTB1 (A24pBPA)-T3 fusion protein was successfully constructed, expressed, and validated via LC-MS (Figure S11). This fusion protein was subsequently conjugated to αmCD205 via P-PIC (Figure 4A, Figure S12), achieving a conjugation efficiency exceeding 80% (Figure S13). Colocalization assays demonstrated that this system significantly increased T3 uptake by RAW264.7 cells (Figure 4B-C).

To assess the therapeutic efficacy of this approach, we utilized an* in vivo* MC38 tumor model overexpressing LMP2A. C57BL/6 mice were subcutaneously inoculated with 1 × 10^6^ MC38/LMP2A cells, with subsequent treatment following the protocol outlined in Figure 3A. Following two rounds of vaccination, tumor growth was significantly suppressed in both the αmCD205-T3_P-PIC_ group and the αmCD205-T3_P-PIC_ + BMS-1 combination group compared with the group receiving a simple mixture of αmCD205 and T3 (Figure 4D), with minimal fluctuations in body weight (Figure 4E). On day 15, splenocytes isolated from treated mice exhibited a marked increase in IFN-γ secretion by both CD8^+^ and CD4^+^ T cells following αmCD205-T3_P-PIC_ and BMS-1 treatment (Figure 4F), indicating enhanced T-cell activation. However, this did not translate into a statistically significant improvement in overall survival between the αmCD205-T3_P-PIC_ + BMS-1 combination group and the αmCD205-T3_P-PIC_ monotherapy group (Figure 4G). Unlike our previous findings on DC-targeted OVA epitope delivery, these results suggested that the EER approach may mitigate limitations associated with the single-antigen epitope approach, such as T-cell activation fatigue and resistance. This highlights its potential to counteract T-cell exhaustion—an issue traditionally addressed through prior administration of PD-1 inhibitors—by inducing a more effective and sustained immune response.

### Application of C-PIC for DC-targeted vaccine antigen delivery

**Figure 5:**
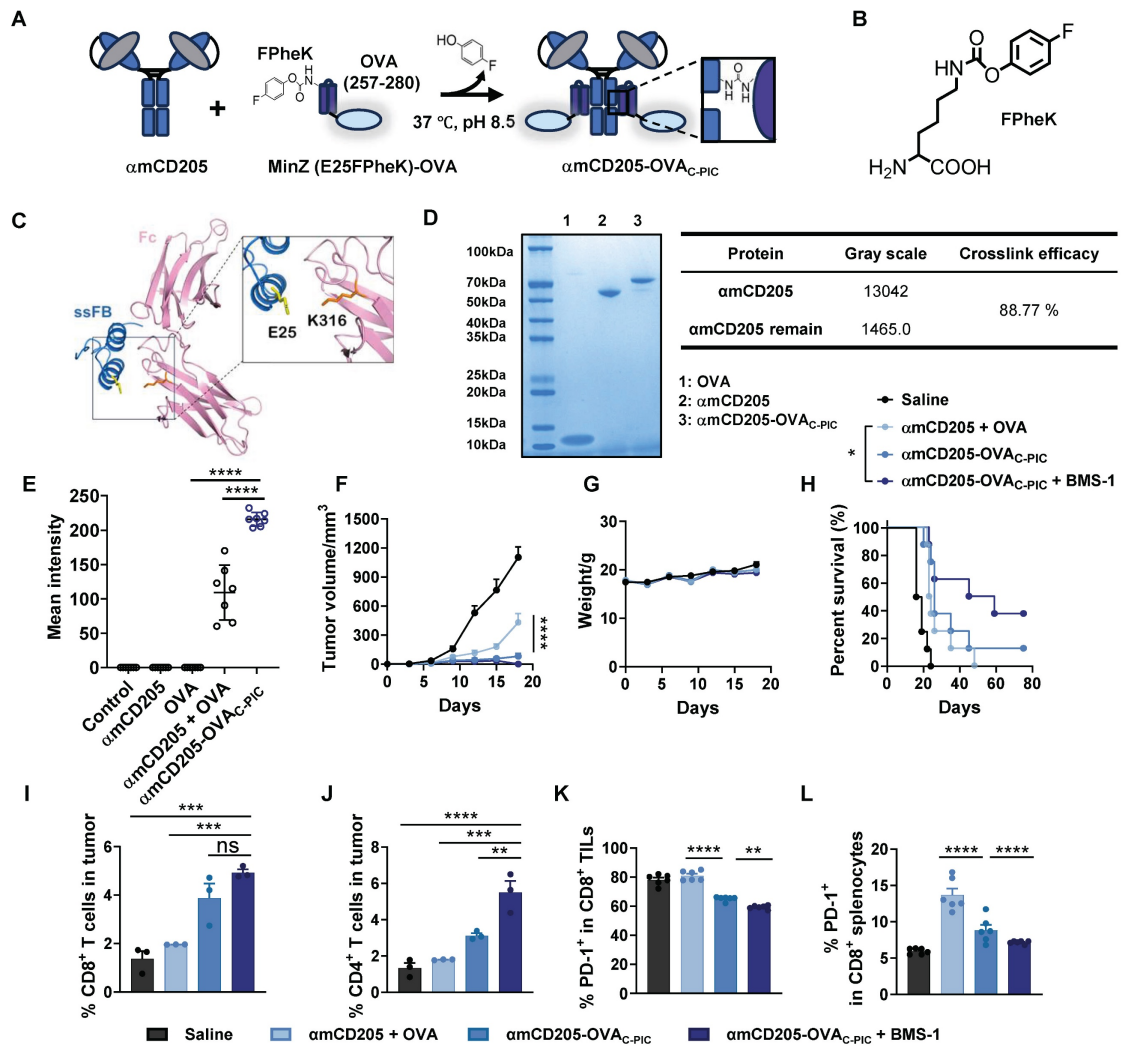
** Design and application of the C-PIC-mediated DC-targeted vaccine.** (A) Schematic representation of the C-PIC procedure. FPheK was incorporated into the MinZ E25 site, and covalent bonding was achieved through mild incubation at 37 °C. (B) Structure of FPheK. (C) Structure of IgG1 Fc fragment interaction with ssFB. PDB file: 1FC2. (D) Reducing SDS-PAGE result of MinZ (E25FPheK)-OVA conjugated to αmCD205. (E) Quantification of the mean integrated fluorescence intensity in the indicated groups (n = 7). (F) Tumor growth curves. (G) Mouse body weight data. (H) Survival curves of the mice treated with the corresponding vaccines or saline. TILs were harvested and analyzed (n = 6). The proportions of (I) CD8^+^ and (J) CD4^+^ TILs are shown (n = 3). (K) Quantification of PD-1 expression levels on CD8^+^ TILs (n = 6). (L) PD-1 expression in CD8^+^ splenocytes was evaluated (n = 6). The data are shown as the means ± SDs. Statistical significance was determined using a one-way ANOVA with Tukey's multiple comparisons test for (E and I-L). For (F), two-way ANOVA with Tukey's multiple comparisons test was used. Survival curve was analyzed with log-rank test (* p < 0.05, ** p < 0.01, *** p < 0.001, **** p < 0.0001).

The effectiveness of P-PIC-mediated antigen delivery has been well established through both *in vitro* and *in vivo* studies. However, the HTB1 peptide (56 amino acids, AAs) poses a potential risk for allergic reactions, necessitating the development of an alternative Fc-binding adaptor. Additionally, UV irradiation can impair antibody functionality, particularly by damaging residues such as tryptophan, tyrosine, phenylalanine, cysteine, and methionine, ultimately leading to a diminished antibody binding activity (Figure S8 E-G, Figure S14A-C) 38. Therefore, optimizing the PIC strategy to enable protein conjugation under milder conditions is critical for maintaining antibody activity and preserving antigen epitope integrity. In our previous work, we developed a C-PIC crosslinker by genetically incorporating 4-fluorophenyl carbamate lysine (FPheK) into the B domain of *Staphylococcus aureus* protein A (FB) 39, using a biorthogonal aminoacyl tRNA synthetase (aaRS)/tRNA pair (Figure 5A-B). Currently, we expressed FB and its two truncated variants, ssFB and MinZ 40, 41, and selected MinZ—an innovative, shorter-length (33 AAs) version of FB with moderate binding affinity (Figure S15A-C)—as the preferred adaptor for the C-PIC strategy. To enable conjugation with αmCD205 under milder conditions, FPheK was site-specifically incorporated at the E25 position of MinZ, a site identified in our previous findings as yielding optimal conjugation efficiency (Figure 5C) 42. The molecular weights of MinZ (E25FPheK)-OVA and wild-type MinZ-OVA were subsequently verified via LC-MS analysis (Figure S17). C-PIC was carried out by incubating MinZ (E25FPheK)-OVA with αmCD205 at a 6:1 molar ratio in PBS (pH = 8.5) at 37 °C for 24 h (Figure 5D). To assess antigen uptake efficiency, we examined the colocalization of the antigen with RAB7, revealing a markedly enhanced colocalization signal in the conjugated group, indicative of superior uptake efficiency (Figure S19A and Figure 5E). Furthermore, αhCD205 was successfully conjugated with MinZ (E25FPheK)-OVA (Figure S20A), and the resulting αhCD205-OVA_C-PIC_ demonstrated significantly great binding affinity to THP-1 cells highlighting the improved efficiency of C-PIC and its potential for DC-targeted vaccine delivery.

### Enhancing the antitumor efficacy of DC-targeted vaccines via C-PIC

The therapeutic potential of C-PIC-mediated DC-targeted vaccine delivery was evaluated in a B16-OVA melanoma model. The experimental groups included the saline, αmCD205 + OVA, αCD205-OVA_C-PIC_, and αmCD205-OVA_C-PIC_ combined with BMS-1 groups, which received the same vaccination and BMS-1 treatments outlined in Figure S21A. Notably, tumor growth was significantly suppressed in the αmCD205-OVA_C-PIC_ + BMS-1 combination group compared with both the saline and αmCD205 + OVA mixture groups, without inducing any noticeable weight loss (Figures 5G). Moreover, mice receiving the combination of the vaccine and ICIs exhibited prolonged survival, with some surviving up to 80 days, underscoring the superior therapeutic efficacy of this approach (Figure 5H). Immunophenotyping of TILs revealed a substantial increase in CD8^+^ and CD4^+^ T cells within tumors from mice treated with the αmCD205-OVA_C-PIC_ + BMS-1 combination treatment (Figure 5I-J). Additionally, PD-1 expression on CD8^+^ TILs was significantly reduced following combination therapy, suggesting enhanced T-cell activation and alleviation of immune exhaustion (Figure 5K). Further analysis of splenic CD8^+^ T cells revealed that BMS-1 administration effectively mitigated immune tolerance by reducing PD-1 expression (Figure 5L), whereas this effect was not observed in tumor-infiltrating or splenic CD4^+^ T cells (Figure S21B-C), highlighting the selective and focused therapeutic action of BMS-1, specifically within the CD8^+^ T-cell population. These findings align with the immune tolerance trend observed in Figure 2G, where the presence of dissociative antigens in the αmCD205 + OVA group contributed to T-cell tolerance, possibly due to prolonged antigen exposure. In addition, the serum OVA-specific antibody titers remained comparable across all the treatment groups, indicating that humoral immunity played a minimal role in tumor suppression in this model (Figure S21D). Collectively, these results highlight the potential of C-PIC-based strategies for optimizing DC-targeted vaccine delivery, providing a promising avenue for enhancing antitumor immunity and improving overall therapeutic efficacy.

### DC-targeted vaccines exhibit a favorable safety profile* in vivo*

**Figure 6:**
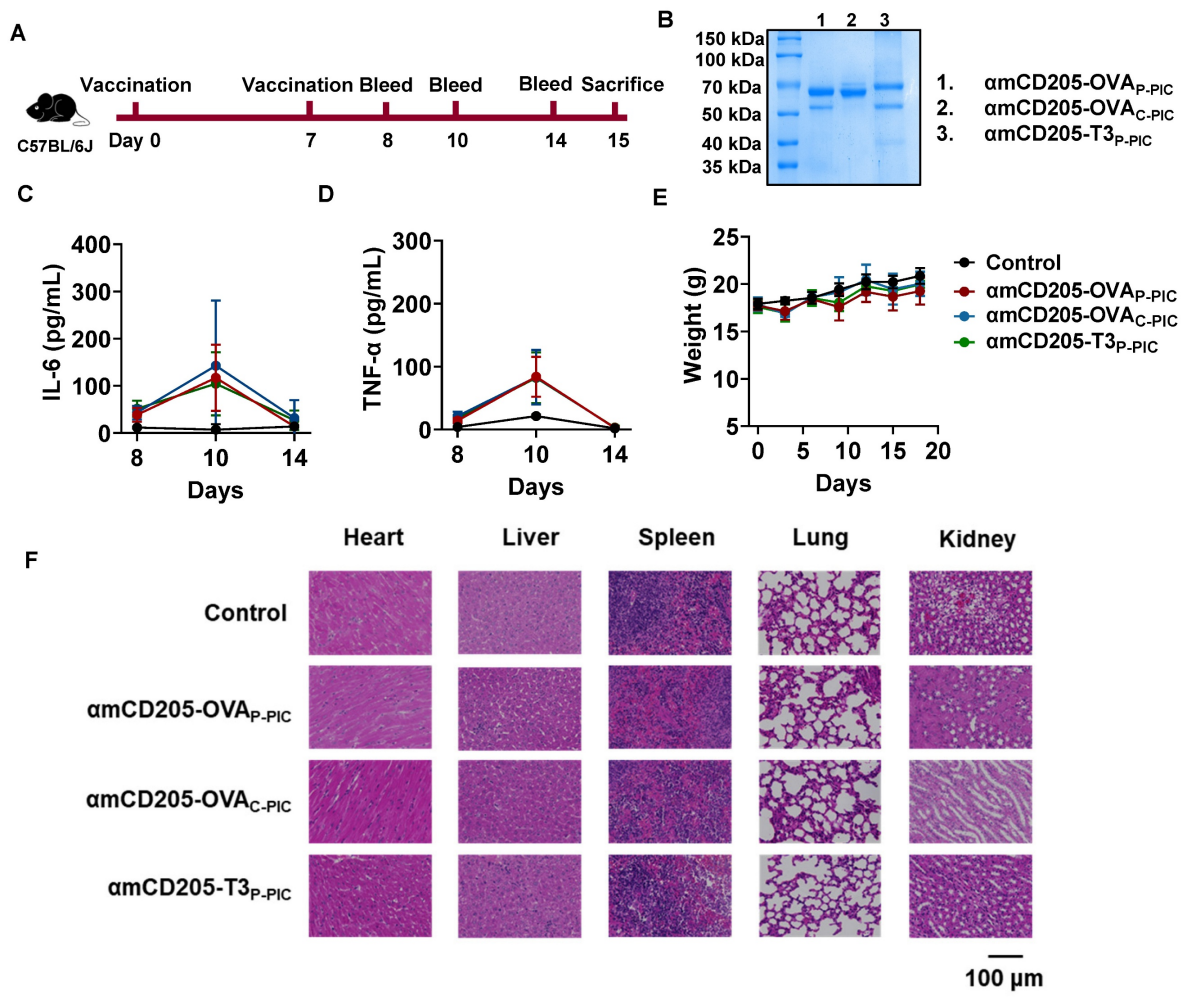
** Safety evaluation of DC-targeted vaccines.** (A) Schematic overview of the safety assessment timeline. Serum samples were collected on days 1, 3, and 7 after the final immunization, and mice were sacrificed on day 8. (B) SDS-PAGE analysis of DC-targeted vaccine formulations. (C-D) Serum levels of IL-6 and TNF-α following immunization (n = 5). (E) Body weight changes of mice after immunization. (F) Representative H&E staining of heart, liver, spleen, lung, and kidney tissues from each group (n = 3). The data are shown as the means ± SDs. Statistical significance was determined using a one-way ANOVA with Tukey's multiple comparisons test (* p < 0.05, ** p < 0.01, *** p < 0.001, **** p < 0.0001).

The *in vivo* safety of the DC-targeted vaccines was assessed in a mouse model. As illustrated in Figure 6A, mice were immunized with amCD205-OVA_P-PIC_, amCD205-OVA_C-PIC_, or amCD205-T3_P-PIC_. Serum samples were collected on days 1, 3, and 7 after the final immunization to evaluate systemic inflammatory responses. A transient elevation of IL-6 and TNF-α was observed on day 3 post-immunization; however, no statistically significant differences were detected when compared with the control group, and cytokine levels returned to baseline by day 7 (Figure 6C-D and Figure S19). Body weight monitoring revealed no significant changes throughout the observation period (Figure 6E). For histopathological analysis, mice were euthanized on day 8 post boost vaccination, and major organs were collected. Hematoxylin and eosin (H&E) staining showed no obvious pathological alterations in the heart, liver, spleen, lung, or kidney in the vaccinated groups compared with the controls (Figure 6F). Collectively, these results indicate that the DC-targeted vaccines did not induce detectable systemic toxicity under the experimental conditions employed in this study.

## Discussion

The development of cancer vaccines remains challenging because of their limited ability to effectively target antigen-presenting cells, resulting in suboptimal immune activation. To overcome this limitation, DC-targeted vaccines incorporating genetically engineered fusion proteins have been designed to enhance antigen delivery and presentation, showing great promise in advancing cancer immunotherapy. To our knowledge, few major studies have yet reported the development of self-assembled DC-targeted vaccines that incorporate tumor-specific neoepitopes using PIC strategies. Importantly, we systematically investigated the development of DC-targeted vaccines with functionally distinct UAAs via distinct PIC approaches and further demonstrated that these vaccines effectively enhanced immune responses against a variety of tumor-specific neoepitopes across different tumor models.

DC-targeted vaccines represent a promising approach in cancer immunotherapy, utilizing specific DC surface receptors to enhance antigen presentation and immune activation 43-45. Among these receptors, CD205 has emerged as a particularly advantageous target compared with CD40, Clec9A, and CD209 owing to its unique ability to activate both CD8^+^ and CD4^+^ T cells 15, 46. This dual activation promotes a more comprehensive and balanced immune response, and the advantages of CD205 as a target are further supported by its broad expression across various DC subsets and its well-documented clinical relevance 47. As a result, we selected CD205 as the target for our DC-directed vaccine. Our *in vivo* studies demonstrated potent CD8^+^ T-cell activation, characterized by increased numbers of TILs and elevated IFN-γ secretion. However, prior studies have indicated that CD205-targeted vaccines may induce immune tolerance in the absence of TLR agonists 48. To overcome this potential limitation, we incorporated a CpG adjuvant to activate TLR9, thereby mitigating the induction of immune tolerance. Importantly, our vaccine exhibited excellent tolerability *in vivo*, with no significant changes in mouse body weight. Following the successful validation of DC targeting using murine anti-CD205 antibodies, we further optimized the platform with a clinically relevant anti-human CD205 monoclonal antibody (clone MG38-3). This optimized vaccine demonstrated a near-saturated binding affinity (> 99%) for hCD205⁺ THP-1 monocytes and efficient antigen uptake via the αhCD205-mediated delivery system. Notably, the transition from anti-murine to anti-human CD205 antibodies was minimally affected by conjugation with UAAs. Collectively, these findings validate the feasibility of translating this DC-targeting strategy into clinical applications, paving the way for further development in cancer immunotherapy.

Traditional DC-targeted vaccine constructs typically rely on the direct fusion of antigens to deliver antibodies, a process that is both time-consuming and costly for gene-based vaccine production. Several protein cross-linking approaches have been developed to achieve high conjugation efficiency, including SpyTag/SpyCatcher, sortagging, and self-labeling nanobodies. However, these methods frequently introduce relatively large exogenous protein sequences, which may increase immunogenicity or compromise antibody functionality 49-51. The PIC method, which leverages latent-bioreactive UAAs, enables site-specific covalent bond formation with proximal target residues 28. Compared with conventional methods, this approach offers superior selectivity and safety. Given its successful application in the development of covalent protein drugs, bispecific antibodies, ADCs, and molecular probes 52, 53, we sought to optimize our DC-targeted delivery system using the PIC strategy. Notably, no studies have reported the application of PIC in the production of antibody-mediated antigen delivery platforms to date. In this study, we preserved the structural integrity of the targeting domain while selectively modifying only the antigen portion using pBPA or FPheK. The conjugation properties of these UAAs facilitated highly efficient and site-specific antigen-antibody conjugation, achieving over 90% efficiency for the OVA epitope and over 80% for the LMP2A EER T3. The PIC method also enables precise control over antigen conjugation sites, maintaining a fixed antibody-to-antigen ratio of 1:2. To further optimize the platform, we selected the moderate-affinity adaptor MinZ to minimize unnecessary immunogenicity and antibody function disruption. Our approach enhances applicability and scalability by streamlining the molecular cloning process into a modular design. This strategy holds significant promise as a highly efficient and controllable method for next-generation vaccine development.

Peptide vaccines have been widely explored in clinical research due to their safety and potential for personalized treatment, and numerous clinical trials have been conducted to assess the efficacy of peptide vaccines in treating glioma, breast cancer, and colorectal cancer 54-56. However, their inherently low immunogenicity necessitates multiple immunizations and high-dose administration to achieve sufficient immune responses, significantly limiting their clinical utility 57. Our previous studies demonstrated that epitope diversity plays a crucial role in vaccine efficacy 37. Compared with single-epitope formulations, vaccines incorporating both B-cell and T-cell epitopes elicit stronger cellular and humoral immune responses, leading to superior tumor suppression. In this study, the LMP2A EER vaccine exhibited distinct advantages. While single-epitope OVA vaccines without ICI coadministration resulted in tumor relapse in the P-PIC assay, the EER T3 vaccine—containing four functional epitopes—achieved an 83% tumor-free survival rate in mice for 75 days, even in the absence of ICI coadministration. Additionally, our previous research revealed that fusion with hEDA and Fc was required to increase the immunogenicity of recombinant EER vaccines and prolong survival. In this study, coupling the vaccine with αCD205 not only improved immune activation and prolonged survival but also streamlined the production of recombinant epitope vaccines, increasing their feasibility for clinical application.

Importantly, despite the promising therapeutic outcomes observed in murine models, the lack of human clinical trials renders our findings preliminary and highlights the gap in clinical translation. The absence of nasopharyngeal carcinoma patient-derived xenografts or orthotopic tumor models raises concerns, as subcutaneous tumor engraftment may not fully capture the vaccine's efficacy against anatomically accurate malignancies 58. Additionally, potential functional perturbations caused by UV exposure and prolonged PIC reactions remain critical considerations. To address these limitations, our future efforts will focus on optimizing conjugation methodologies to develop an improved PIC strategy with enhanced efficiency, short reaction times, and minimal impact on antibody functionality. Moreover, the incorporation of UAAs introduces the potential risk of neo-antigen formations, which remains an important concern. Previous studies have shown that the integration of pNO_2_Phe, SO_3_Tyr, and _3_NO_2_Tyr into native proteins can disrupt immune tolerance and elicit humoral immune response against the modified proteins 59. The unique side chains of UAAs may further break immune-tolerance to endogenous proteins in a T cell-dependent manner, generating antibodies responses that are not restricted to epitopes containing the unnatural residues. Therefore, comprehensive safety and immunogenicity evaluations will be essential before advancing this strategy toward clinical translation.

## Conclusion

This study introduces a modular design for a therapeutic cancer vaccine composed of two key components: a DC-targeted domain and antigen cargo. The DC-targeted domain employs an anti-CD205 antibody that specifically recognizes and delivers antigen cargo into DCs. The antigen cargo is modified with an antibody affinity peptide incorporating UAAs to enable photoreactive or chemically reactive PIC. These components form a covalent bond, allowing for the rapid preparation of DC-targeted vaccines within hours. The effectiveness of the DC-targeted delivery system has been validated through both *in vitro* and *in vivo* studies, which demonstrated efficient antigen uptake and significant tumor inhibition across various models. Overall, our design, with its modular structure and proven efficacy, offers a promising and adaptable platform for developing therapeutic cancer vaccines underscoring its potential for broad clinical applications and shedding light on next-generation cancer immunotherapies.

## Materials and Methods

### Cell lines and culture conditions

The mouse macrophage cell line RAW264.7 and the human monocyte cell line THP-1 were purchased from American Type Culture Collection (ATCC) and cultured in RPMI-1640 medium (HyClone, SH30255.01) containing 10% Fetal Bovine Serum (FBS), 1% penicillin-streptomycin (PS), 2 mM L-glutamine (Gln), 1 mM sodium pyruvate (SP) and 0.1 mM non-essential amino acids (NEAA). The B16-OVA cell line was kindly provided by Demin Zhou (Peking university), and the MC38-LMP2A cell line was generated via lentivirus transduction. These cell lines were maintained in high glucose DMEM which was supplemented with 10% FBS, 1% PS, 2 mM Gln, 1 mM SP and 0.1 mM NEAA. The Freestyle293 cell line (293F) was obtained from Sinobiological and cultured in SMM 293-TII expression medium (Sinobiological, M293TII).

### Chemicals

p-benzoyl-L-phenylalanine (pBPA) was purchased from MATEK (Suzhou, China). BMS-1 was purchased from Selleck (S7911).

4-fluorophenyl carbamate lysine (FPheK) was synthesized as previously reported (Figure S16A)^27^. Briefly, Boc-L-Lys-OH (2 g, 8.12 mM) was dissolved in DCM (30 mL). The reaction mixture was cooled to 0 ^°^C, TEA (2.83 mL, 2.5 equiv.) and 4-fluorophenyl chloroformate (1.12 mL, 1.05 equiv.) were slowly added sequentially via a syringe. The mixture was stirred at room temperature (RT) for 16 h. The mixture was diluted by cold H_2_O (30 mL), the pH was adjusted to 3 with HCl (1.0 M aqueous solution). Layers were separated, and the aqueous phase was further washed by DCM (20 mL). The combined organic solutions were dried with Na_2_SO_4_ (anhydrous), filtered and concentrated in *vacuo*. The residue was purified by flash column chromatography on silica (EA: Hexane = 1: 1) to give the desired intermediate.

The above intermediate was dissolved in DCM (20 mL), after the addition of TFA (5 mL), the solution was stirred at RT and monitored by TLC. 5 h later, the reaction mixture was evaporated, the product was dried under vacuum to afford FPheK (1.0 g, 31%) as light-yellow oil. The analytical data are consistent with the literature reports (Figure S16B-C).

### Molecular cloning

Plasmids used in this work were designed and constructed via standard homologous recombination protocols. The HTB1-OVA (257-280) and MinZ-OVA (257-280) constructs were synthesized by Genewiz, Inc. (Suzhou, China). The PCR-amplified products were subsequently cloned and inserted into the pET32a and pET22b vectors. A His tag was appended to the C-terminus of both HTB1-OVA (257-280) and MinZ-OVA (257-280) to facilitate purification. For the T3 construct, the HTB1-T3 gene was synthesized by Genewiz, Inc. and fused with MBP tag via overlapping PCR. A thrombin cleavage site and a Flag tag were introduced between MBP and HTB1-T3, and a His tag was added at the C-terminus of T3. To generate the UAA-incorporated plasmid, site-directed mutagenesis was performed to introduce an amber mutation at the HTB1 residues (A24X or K28X) or MinZ residue (E25X). For CD205-scFv, the VL and VH sequences were derived from the clone NLDC-145 or MG38-3 and linked by a (G_4_S)_3_ flexible linker. The LH and HL orientations were synthesized by Genewiz, Inc. The final construct was inserted into the pCAGGS vector between the Nhe I and Not I restriction sites. For the pCAGGS-αCD205 plasmid, the scFv sequence was codon optimized for eukaryotic expression and fused with the hIgG1 Fc fragment. The resulting construct was inserted into the pCAGGS vector. Similarly, the αhCD205 construct was synthesized by Genewiz, Inc. and cloned and inserted into pCAGGS following the same method.

### Protein expression and purification

HTB1-OVA and MinZ-OVA were expressed using BL21 (DE3). The corresponding plasmids were transformed into competent cells. The strains were inoculated into 2YT medium and 0.1 mM IPTG was added when OD_600_ reached 0.8-1.0. The cells were left grown at a condition of 18 ℃, 180rpm for 16 h. Proteins were purified using Ni beads (Solarbio, P2010). HTB1 (A24pBPA)-OVA, HTB1 (K28pBPA)-OVA, and MinZ (E25FPheK)-OVA were expressed by cotransforming pEVOL-pBPA or pUltra-FPheKRS with the appropriate plasmids and culturing with 1 mM pBPA or FPheK. The purification process for these proteins was identical to that used for the wild-type proteins. The expression of MBP-Flag-HTB1 (A24pBPA)-T3 His involved a two-step affinity chromatography process. Ni beads were used to bind intact protein (excluding the truncated forms). 8 IU/mg thrombin was added to remove MBP tag, and anti-DYKDDDDK affinity resin (SinoBiological, 101274) was further used to purify the T3-based fusion proteins.

The αmCD205 and αhCD205 fusion proteins were expressed in 293F suspension cells using their respective vectors via transient transfection. Briefly, transfection was performed when the 293F cell concentration reached 3 × 10^6^ cells/mL. A defined amount of plasmid was mixed with PEI MAX (Polysciences, Inc.) at a 1:2.5 mass/volume ratio. After the mixture was incubated for 30 min, it was added to the cell suspension. Culture medium was harvested 72 h posttransfection. Proteins were purified using Protein G affinity chromatography, and verified via SDS-PAGE.

### PIC

To perform P-PIC, αmCD205 or αhCD205 was dissolved in PBS at a final concentration of 10 μM and mixed with HTB1 (A24pBPA)-OVA or HTB1 (A24pBPA)-T3 at a 1:8 molar ratio. The mixture was immediately placed on ice and subjected to UV irradiation at 365 nm for 2 h using a UV crosslinker (UV07-II, MCGS). This process induces covalent bond formation between the interacting proteins subjected to UV radiation, allowing for precise and controlled conjugation. Following irradiation, P-PIC products were purified with HPLC to remove extra OVA or T3 peptides.

To perform C-PIC, αmCD205 or αhCD205 was mixed with MinZ (E25FPheK)-OVA at a 1:6 molar ratio, followed by incubation at 37 °C for 24 h to facilitate the interaction between the two proteins. The pH of the reaction mixture was then adjusted to 8.5 to optimize the conditions for efficient conjugation. The C-PIC technique relies on close spatial interactions between proteins, resulting in the formation of stable covalent conjugates. Unlabeled peptides were removed by protein G affinity chromatography.

All the PIC samples were analyzed by reducing SDS-PAGE to assess the efficiency of conjugation. The resulting gel was stained with Coomassie Brilliant Blue to visualize the protein bands and evaluate the extent of conjugation on the basis of the shift in the molecular weights of the proteins.

### LC‒MS analysis

The molecular weights of the proteins were measured using LC-MS. Briefly, protein samples were desalted by ultrafiltration. 1 mM DTT was added for reducing condition analysis. The molecular weight was determined via Q Exactive HF-X mass spectrometer (Thermo Fisher Scientific) and data was performed via BioPharma Finder 3.2 software.

### ELISA

The binding affinity of Fc and FB or its variants was evaluated using ELISA. The 96-well ELISA plates were coated with Fc protein, followed by blocking with 5% BSA-PBS. The serial diluted adaptors were added to the wells, and the plates were incubated for 2 h at RT. This was followed by the additional of an HRP-labeled anti His tag antibody and a similar round of incubation. Following washing, TMB (Elabscience, E-IR-R201) was added to develop color reaction. The color change was detected via Cytation 5 Cell Imaging Reader (Agilent) with an excitation wavelength of 450 nm.

### Cell based ELISA

3×10^4^ RAW264.7 or THP-1 cells were seeded into a ploy-D-lysine (Sigma‒Aldrich, 27964-99-4) precoated 96-well plate. 24h later, cells were fixed with 4% PFA (Biosharp, BL539A) for 15min. After that, cells were incubated with 0.1 M Glycine-PBS and 3% H_2_O_2_-PBS successively and blocked with 2% BSA-PBS. Corresponding protein constructs were diluted and added into a final volume of 100μL each well. After washing, cells were incubated with HRP-labeled anti-DYKDDDDK antibody (MF085). The color reaction was then initiated by adding TMB substrate. The reaction was stopped with 2 M H_2_SO_4_, and the absorbance was measured at 450 nm.

### Immunofluorescence colocalization

The poly-D-lysine precoated slides were placed into a 12-well plate, and 0.5×10^6^ RAW264.7 or THP-1 were seeded. 24 h later, 100 nM vaccine constructs were added, and cells were incubated for an additional hour. After incubation, cells were fixed with 4% PFA, permeabilized with 0.25% Triton X-100-PBS (Sangon Biotech, 9002-93-1), and blocked with dilution buffer (PBST with 1% BSA and 0.1M Glycine). Cells were incubated with antibodies mixture including Alexa Fluor 647 labeled-Anti RAB7 (Abcam, ab198337) and Alexa Flour 488 labeled-Anti DYKDDDDK (Proteintech, CL488-80010). Lastly, the nuclei were stained using 10 μg/mL Hoechst (Solarbio, C0031). Fluorescent images were captured using Nikon A1R HD25 confocal microscope.

### Flow cytometry

The cells were incubated with the corresponding protein mixture and fluorescent dyes labeled antibodies respectively for 1 h on ice. After washing, the stained cells were analyzed via a Thermo Fisher Attune NxT flow cytometer (Thermo Fisher Scientific, A29001). Data was analyzed via FlowJo 10 software.

### Animal study

Animal experiment procedures were approved by the Peking University Shenzhen graduate School Animal Care and Use Committee and performed according to the national and international ethical guidelines. Six-week-old C57BL/6J mice were purchased from Zhejiang Vital River Laboratories Animal Technology Co., Ltd. B16-OVA cells (0.5 × 10^6^) or MC38-LMP2A cells (1 × 10^6^) were subcutaneously inoculated into the right flanks of the mice. One day after tumor inoculation, 10 μg of vaccine was injected intraperitoneally along with 50 μg of CpG 1826 (MCE, 202668-42-6) in 100 μL of PBS. The CpG1826 sequence was d(P-thio) (T-C-C-A-T-G-A-C-G-T-T-C-C-T-G-A-C-G-T-T) and no physical linkage was presented between CpG and vaccine. The molar amounts of the other control groups were adjusted to match the 10 μg dose used in the conjugation group. A booster vaccination was administered 7 days after the prime dose. As for the combination of checkpoint inhibitors, 50 μg/kg BMS-1 (Selleck.cn, S7911) was administered via intraperitoneal injection two days after immunization. Mouse body weight and tumor volume were measured every three days.

### TIL detection

To analyze TILs, tumor tissue was harvested and minced into small pieces. The minced tissue was then digested with collagenase IV, hyaluronidase, and DNase I for 2 h on a shaking platform. After digestion, the tissue was filtered through a strainer to remove debris, and the resulting cell suspension was adjusted to a density of 1-5 × 10^6^ cells/mL for flow cytometry analysis. TILs were identified using PE-conjugated anti-mouse CD3ε (BioLegend, 100308) and FITC-conjugated anti-mouse CD8α (BioLegend, 100706) antibodies. The expression of PD-1 on TILs was detected using Brilliant Violet 421-conjugated anti-mouse PD-1 antibodies (BioLegend, 135217).

### ELISpot

Mice were euthanized 7 days after the boost vaccination. Splenocytes were isolated after homogenization and red blood cells lysed (RBC lysis buffer, Biolegend, 420302). The release of IFN-γ under antigen stimulation was detected using mouse IFN-γ ELISpot precoated Kit (DKW22-2000). Briefly, 4×10^5^ slenocytes were seeded into each well and stimulated with the corresponding antigen (HTB1-OVA, HTB1-T3 or MinZ-OVA) at a concentration of 10 µg/mL for 48 h. After incubation, the spots were developed according to the manufacturer's instructions.

### Intracellular cytokine staining

4×10^6^ Splenocytes were seeded into a 24-well plate and stimulated with corresponding antigen including HTB1-OVA, HTB1-T3 and MinZ-OVA at a concentration of 10 μg/mL. The antigen stimulation lasted for 24h, with Brefelsin A added during the last 5h. The collected cells were first stained with PE-anti-mouse CD3ε and FITC-anti-mouse CD8a antibodies. Subsequently, cells were fixed, permeabilized and stained with APC-Anti mouse IFN-γ. The antigen stimulated secretion of IFN-γ was evaluated via flow cytometry.

### Serum antibody titer assay

Blood was collected via the orbital vein, and the serum was isolated by centrifugation. A 96-well ELISA plate was coated with 10 µg/mL intact ovalbumin and subsequently blocked with 5% BSA -PBS. A series of diluted serum samples were then added, and followed by 2h incubation at RT. HRP-labeled anti-mouse antibody was added, and the plates were incubated for a similar round of incubation. After washing, the color reaction was developed by adding TMB, and the absorbance was measured.

### Statistical analysis

Statistical analysis was performed using GraphPad Prism 10 software with one- or two-way ANOVA followed by Tukey's multiple comparisons test. Statistical significance was considered when the p value was less than 0.05. The data are presented as the means ± standard deviations (SDs) from at least three biological replicates.

## Supplementary Material

Supplementary information, figures and tables.

## Abbreviations

UAA: unnatural amino acid
pBPA: p-benzoyl-L-phenylalanine
FPheK: 4-fluorophenyl carbamate lysine
PIC: proximity-induced conjugation
OVA: Ovalbumins
DC: dendritic cell
TIL: tumor infiltration lymphocyte
PD-1: Programmed cell death protein
EER: epitope enrichment region
